# Wnt5a causes ROR1 to complex and activate cortactin to enhance migration of chronic lymphocytic leukemia cells

**DOI:** 10.1038/s41375-018-0306-7

**Published:** 2018-12-19

**Authors:** Md Kamrul Hasan, Laura Rassenti, George F. Widhopf, Jian Yu, Thomas J. Kipps

**Affiliations:** Moores Cancer Center, University of California San Diego, La Jolla, CA USA

**Keywords:** Chronic lymphocytic leukaemia, Translational research

## Abstract

Chronic lymphocytic leukemia cells (CLL) migrate between the blood and lymphoid tissues in response to chemokines. Such migration requires structured cytoskeletal-actin polymerization, which may involve the protein cortactin. We discovered that treatment of CLL cells with Wnt5a causes Receptor tyosin kinase-like orphan receptor 1 (ROR1) to bind cortactin, which undergoes tyrosine phosphorylation at Y421, recruits ARHGEF1, and activates RhoA, thereby enhancing leukemia-cell migration; such effects could be inhibited by cirmtuzumab, a humanized mAb specific for ROR1. We transfected the CLL-cell-line MEC1 with either full-length ROR1 or various mutant forms of ROR1 to examine the structural features required for binding cortactin. We found that the proline-rich domain (PRD) was necessary for ROR1 to recruit cortactin. We generated MEC1 cells that each expressed a mutant form of ROR1 with a single amino-acid substitution of alanine (A) for proline (P) in potential SH3-binding sites in the ROR1-PRD at positions 784, 808, 826, 841, or 850. In contrast to wild-type ROR1, or other ROR1^P=>A^ mutants, ROR1^P(841)A^ failed to complex with cortactin or ARHGEF1 in response to Wnt5a. Moreover, Wnt5a could not induce MEC1-ROR1^P(841)A^ to phosphorylate cortactin or enhance CLL-cell F-actin polymerization. Taken together, these studies show that cortactin plays an important role in ROR1-dependent Wnt5a-enhanced CLL-cell migration.

## Introduction

Receptor tyrosine kinase-like orphan receptor 1 (ROR1) is a highly conserved surface protein that ordinarily functions in the organogenesis of skeletal and neural tissues [1–4]. The expression of ROR1 attenuates during fetal development [5], becoming negligible on most tissues at term [6]. However, we and others have found that ROR1 distinctively is expressed on the leukemia cells of most patients with chronic lymphocytic leukemia (CLL) [6–8], suggesting that it may play a pathogenic role. Consistent with this concept are studies demonstrating that high-level leukemia-cell expression of ROR1 can enhance disease progression in mouse models [9], and associate with a shorter median time from diagnosis to initial therapy and shorter survival for patients with CLL [10].

We described that Wnt5a could act as a ligand for ROR1 [6], which prior studies showed could enhance directional cell migration and planar-cell polarity by inducing non-canonical Wnt signaling [11]. In more recent studies on CLL cells, we described that Wnt5a could induce ROR1 to recruit and activate guanine exchange factors (GEFs), and thereby enhance leukemia-cell migration and proliferation [12]. These effects of Wnt5a on CLL cells could be inhibited by cirmtuzumab (UC-961), a humanized mAb specific for ROR1. However, the molecular mechanism(s) for how ROR1 exerts these effects and how cirmtuzumab could inhibit such outcomes are still unclear.

Cortactin (also called EMS1, or CTTN) plays an important role in the reorganization of the cytoskeleton required for migration and planar-cell polarity. Cortactin is a cytoplasmic protein that can promote polymerization and rearrangement of the actin cytoskeleton required for cell migration upon its tyrosine phosphorylation or activation [13–15]. Cortactin contains a SH3 domain, which allows it to bind in the proline-rich-domain (PRD) of other proteins at characteristic SH3-binding motifs (-P-X-X-P-) [16–18]. Cortactin also is expressed in CLL cells and the CLL-cell-line MEC1 [19, 20]. Moreover, expression and phosphorylation of cortactin correlate with enhanced CLL-cell migration [19, 21]. However, the role of Wnt5a in the phosphorylation of cortactin was unknown. Here we report our studies evaluating whether cortactin is recruited to ROR1 upon stimulation with Wnt5a, undergoes tyrosine phosphorylation, and recruits/activates ARHGEF1 to promote F-actin polymerization and leukemia-cell migration.

## Materials and methods

### Cell culture and CLL specimens

MEC1 cells were cultured in RPMI medium with 10% FBS, 1% penicillin/streptomycin and maintained at 37 °C in a humidified atmosphere of 5% CO_2_, and tested negative for mycoplasma contamination. Media and supplements were purchased from Life Technologies (Carlsbad, CA, USA). Blood samples were collected from CLL patients at the Moores Cancer Center. Peripheral blood mononuclear cells were isolated by density centrifugation with Ficoll-Paque PLUS (GE Healthcare Life Sciences) and suspended in 90% FBS (Omega Scientific) and 10% DMSO (Sigma-Aldrich) for viable storage in liquid nitrogen.

### Statistical analysis

Data are presented as mean ± SD. Differences between two groups were determined by unpaired two-tailed Student’s *t*-test. Differences between multiple groups were determined by one-way ANOVA with post-hoc Tukey HSD test. All *P* values of less than 0.05 were considered significant. Analysis for significance was performed with GraphPad Prism 6.0 (GraphPad Software Inc.).

## Results

### Tyrosine phosphorylation of cortactin is higher in ROR1^Pos^ CLL

**Fig. 1:**
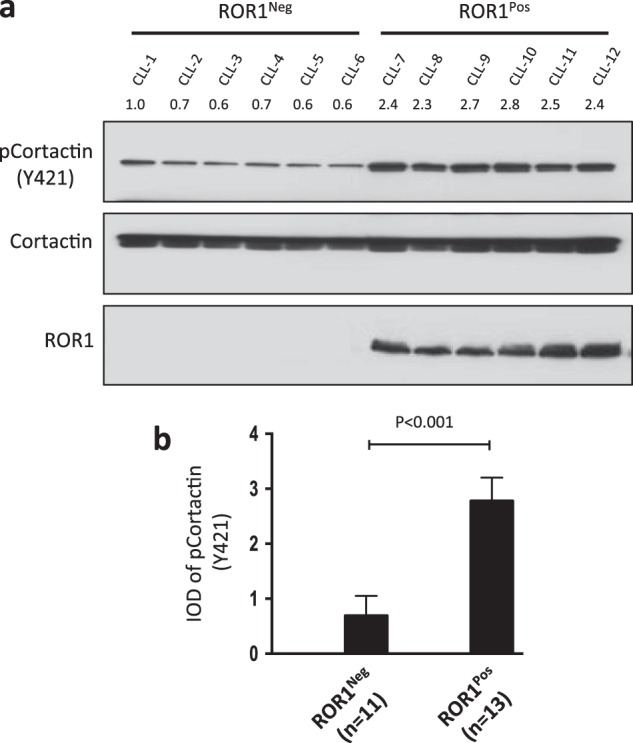
Phosphorylation of cortactin is high in ROR1^Pos^ CLL. **a** Immunoblot analysis of lysates prepared from primary ROR1^Pos^ or ROR1^Neg^ CLL cells of different patients; filters were probed with anti-cortactin, anti-phospho-cortactin (anti-pCortactin (Y421)), or anti-ROR1 antibody, as indicated on the left. The numbers above the top lane are ratios of band integrated optical density (IOD) of phosphorylated versus total cortactin. **b** Phosphorylation of cortactin (at Y421) was assessed by immunoblot analysis of lysates prepared from primary CLL cells of different patients with CLL cells that did (ROR1^Pos^ (*n* = 13)) or did not (ROR1^Neg^ (*n* = 11)) express ROR1. The ratios of band IOD of phosphorylated versus total cortactin were determined and plotted in the graph. Data are shown as mean ± SD. *P* < 0.001, as assessed by two-tailed Student’s *t-*test

Prior studies found that cortactin may be constitutively phosphorylated at Y421 in freshly isolated CLL cells [21]. Since other studies found that ROR1 is variably expressed on the CLL cells of different patients [22], we examined for expression of cortactin and phosphorylated cortactin in CLL cells that expressed ROR at high levels (ROR1^Pos^ CLL) versus low-to-negligible levels (ROR1^Neg^ CLL). We found that the amount of cortactin did not vary between such samples (Fig. 1a, upper panel). However, the mean level of cortactin that was phosphorylated at Y421 (pCortactin) and the ratio of pCortactin/cortactin were significantly higher in ROR1^Pos^ CLL (*n* = 13) than in ROR1^Neg^ CLL cells (*n* = 11) (*P* < 0.001) (Fig. 1a, b).

### Wnt5a induces ROR1/cortactin association in primary CLL cells

**Fig. 2:**
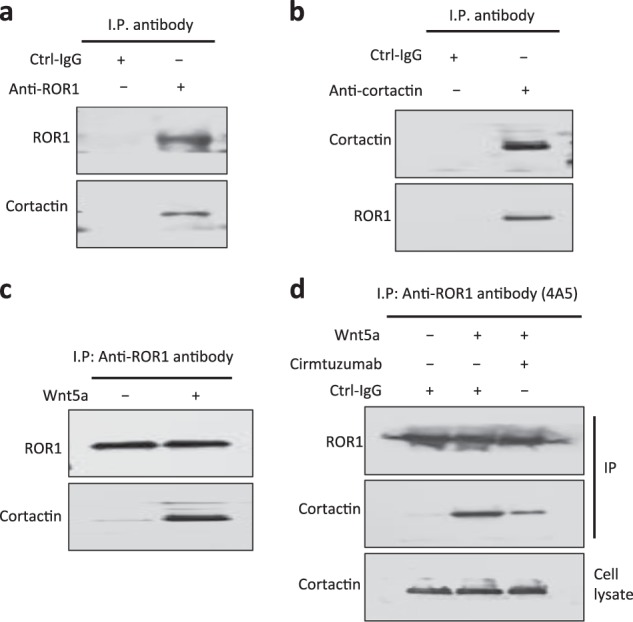
Association of ROR1 with cortactin in primary CLL cells. **a** Immunoblot analysis of anti-ROR1 ip or control IgG (Ctrl-IgG) ip, as indicated at the top, using lysates prepared from freshly isolated primary CLL cells; the filters were probed with anti-ROR1 or anti-cortactin antibody, as indicated on the left. **b** Immunoblot analysis of anti-cortactin ip or Ctrl-IgG ip, as indicated at the top, using lysates prepared from freshly isolated primary CLL cells; the filters were probed with anti-ROR1 or anti-cortactin antibody, as indicated on the left. **c** Immunoblot analysis of anti-ROR1 ip using lysates prepared from overnight, serum-starved primary CLL cells that subsequently were treated for 30 min without (–) or with (+) Wnt5a (100 ng/ml), as indicated on the top; the filters were probed with anti-ROR1 or anti-cortactin antibody, as indicated on the left. **d** Immunoblot analysis of anti-ROR1 ip, as indicated at the top, using lysates prepared from serum-starved primary CLL cells that had been treated with cirmtuzumab, without (–) or with (+) Wnt5a (100 ng/ml); filters were probed with anti-ROR1 or anti-cortactin antibody, as indicated on the left. An immunoblot of the whole-cell lysates (“Cell lysate”) of the CLL cells treated without or with cirmtuzumab and probed with anti-cortactin mAb is provided in the bottom panel

We performed immunoblot analysis of anti-ROR1 or anti-cortactin ip and found that ROR1 complexed with cortactin in freshly isolated primary CLL cells (Fig. 2a, b). However, this complex was not apparent in CLL cells that were cultured overnight in media lacking Wnt5a. When we examined serum-starved CLL cells that were cultured for 30 min in complete media without or with exogenous Wnt5a, we found that the Wnt5a-treated CLL cells again had ROR1 complexed with cortactin (Fig. 2c). Treatment of CLL cells with the ant-ROR1 antibody cirmtuzumab could block the capacity of Wnt5a to induce cortactin to complex with ROR1, as assessed in immunoblot analysis of ip generated from treated CLL cells using an anti-ROR1 mAb (4A5) specific for a different epitope than that recognized by cirmtuzumab (Fig. 2d).

### Wnt5a induces ROR1-dependent cortactin phosphorylation and enhances CLL-cell migration

**Fig. 3:**
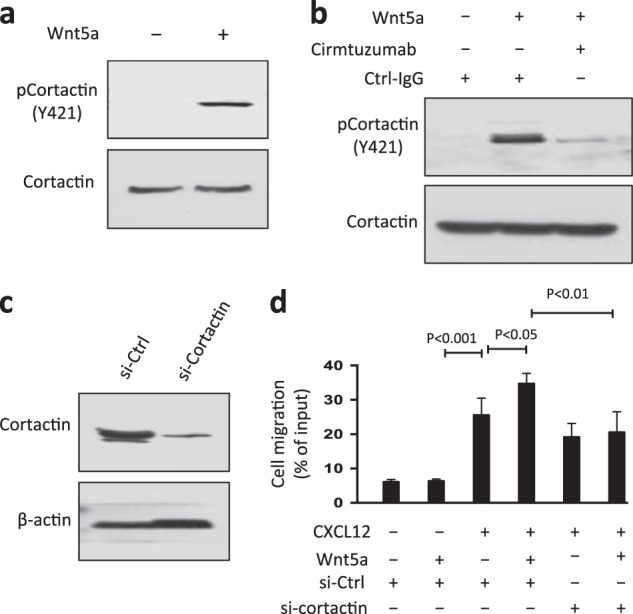
Wnt5a induces ROR1-dependent phosphorylation of cortactin and enhances chemokine-directed leukemia-cell migration. **a** Immunoblot analysis of lysates prepared from overnight, serum-starved primary CLL cells that subsequently were treated for 5 min without (–) or with (+) Wnt5a (100 ng/ml), as indicated on the top; the filters were probed with anti-cortactin or anti-pCortactin (Y421) antibody, as indicated on the left. **b** Immunoblot analysis of lysates prepared from overnight, serum-starved primary CLL cells that subsequently were treated with Ctrl-IgG or cirmtuzumab (10 μg/ml), without (–) or with (+) Wnt5a (100 ng/ml), as indicated on the top; the filters were probed with anti-cortactin or anti-pCortactin (Y421) antibody, as indicated on the left. **c** Immunoblot analysis of lysates prepared from CLL-cells transfected 72 h before with control siRNA or siRNA targeting cortactin; filters were probed with anti-cortactin or anti-β-actin antibody, as indicated on the left. Cell viability was over 85% in control-and cortactin-siRNA transfected cells. **d** CLL-cell migration in response to CXCL12 (200 ng/ml) was assessed without (–) or with (+) exogenous Wnt5a (200 ng/ml), as indicated at the bottom. Data are shown as mean ± SD from three independent experiments of CLL cells from each of six patients. *P* < 0.05; *P* < 0.01; *P* < 0.001, as assessed by two-tailed Student’s *t-*test

We cultured CLL cells in media lacking Wnt5a and observed attrition in the level of phosphorylated cortactin over time (Supplementary Figure S1). Treatment of Wnt5a-starved CLL cells with exogenous Wnt5a for 5 min induced tyrosine phosphorylation of cortactin (Fig. 3a). However, pre-treatment of CLL cells with cirmtuzumab blocked the ability of Wnt5a to induce tyrosine phosphorylation of cortactin (Fig. 3b), indicating that Wnt5a induced tyrosine phosphorylation of cortactin in a ROR1-dependent manner.

Previously we reported that CLL cells treated with Wnt5a could enhance migration directed by chemokines such as CXCL12, and cirmtuzumab could inhibit such effects [12, 23]. We found that treatment of CLL cells with cortactin-specific siRNA, but not non-specific siRNA, could reduce expression of cortactin and suppress the capacity of Wnt5a to enhance chemokine-directed CLL-cell migration (Fig. 3c, d). We also examined the motility of CLL-cells co-cultured with marrow mesenchymal stromal cells (MSCs) cultured at a physiologic oxygen tension (e.g., 5% O_2_ in N_2_). Such MSCs secrete CXCL12 or Wnt5a, as described [24, 25]. We found that co-culture with stromal cells enhanced the migration capacity of CLL cells. However, silencing the expression of cortactin with specific siRNA, or treatment with anti-ROR1 antibody, could significantly inhibit the motility of CLL cells co-cultured with stromal cells, indicating that ROR1/cortactin-signaling plays an important role in CLL-cell migration (Supplementary Figures S2 A and B).

Previously we described that HS1 contributed to the capacity of Wnt5a to enhance leukemia-cell migration [23]. Here we found that silencing both cortactin and HS1 had an additive effect in inhibiting Wnt5a-induced chemokine-directed migration of CLL cells. Moreover, the inhibitory effect of silencing both cortactin and HS1 was significantly greater than that caused by silencing either cortactin or HS1 alone (Supplementary Figure S3).

### ROR1 associates with cortactin, which undergoes tyrosine phosphorylation in MEC1-ROR1 cells

**Fig. 4:**
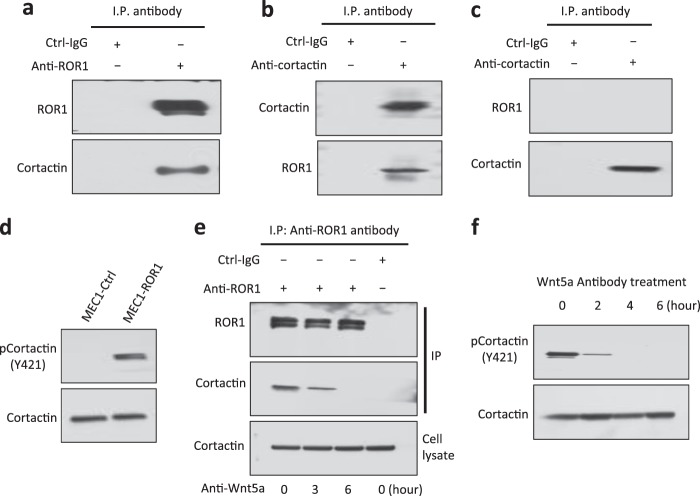
ROR1 in MEC1-ROR1 cells associates with cortactin, which undergoes Wnt5a-dependent phosphorylation at Y421. **a** Immunoblot analysis of anti-ROR1 ip or Ctrl-IgG ip, as indicated at the top, using lysates prepared from MEC1-ROR1 cells; the filters were probed with anti-ROR1 or anti-cortactin antibody, as indicated on the left. **b** Immunoblot analysis of anti-cortactin ip or Ctrl-IgG ip, as indicated at the top, using lysates prepared from MEC1-ROR1 cells; filters were probed with anti-ROR1 or anti-cortactin antibody, as indicated on the left. **c** Immunoblot analysis of anti-cortactin ip or Ctrl-IgG ip, as indicated at the top, using lysates prepared from the ROR1-negative cell line, MEC1; the filters were probed with anti-ROR1 or anti-cortactin antibody, as indicated on the left. **d** Immunoblot analysis of lysates prepared from MEC1 or MEC1-ROR1 cells, as indicated on the top; filters were probed with anti-cortactin, anti-pCortactin (Y421), or ROR1 antibody, as indicated on the left. **e** Immunoblot analysis of anti-ROR1 ip or Ctrl-IgG ip, as indicated at the top, using lysates prepared from MEC1-ROR1 cells that had been treated with a Wnt5a neutralizing antibody (2 µg/ml, R&D, Cat#MAB645) for the times indicated at the bottom (in hours); filters were probed with anti-ROR1 or anti-cortactin antibody, as indicated on the left. An immunoblot of the whole-cell lysates of the MEC1-ROR1 cells treated with the Wnt5a neutralizing antibody and probed with an anti-cortactin mAb is provided in the bottom panel. **f** Immunoblot analysis of lysates prepared from MEC1-ROR1 cells that had been treated with a Wnt5a neutralizing antibody (2 µg/ml, R&D) for the times indicated at the top (in hours); filters were probed with anti-cortactin, anti-pCortactin (Y421), as indicated on the left. An immunoblot of the whole-cell lysates of the MEC1-ROR1 cells treated with the Wnt5a neutralizing antibody and probed with anti-cortactin mAb is provided in the bottom panel

MEC1 is a cell-line derived from CLL cells that has been used as a cell model system for CLL [26]. In prior studies, we found that MEC1 does not express ROR1, but expresses high levels of Wnt5a [12]. We transfected MEC1 cells with a ROR1-expression plasmid to generate MEC1-ROR1 cells, which express human ROR1. We performed immunoblot analysis of anti-ROR1 or anti-cortactin ip and found that ROR1 associated with cortactin in MEC1-ROR1 cells (Fig. 4a, b). As expected, ROR1 was not detected in the anti-cortactin ip using lysates of MEC1 cells lacking ROR1 (Fig. 4c). Consistent with the dependency of cortactin phosphorylation on ROR1, we found that MEC1-ROR1 cells showed higher-levels of phosphorylated cortactin than MEC1 cells, which had negligible levels of phosphorylated cortactin (Fig. 4d). Treatment of MEC1-ROR1 cells with neutralizing antibodies to Wnt5a caused ROR1 to dissociate from cortactin (Fig. 4e), and reduced the levels of phosphorylated cortactin in a time-dependent manner (Fig. 4f).

### ARHGEF1 associates with ROR1/cortactin and enhances activation of RhoA

**Fig. 5:**
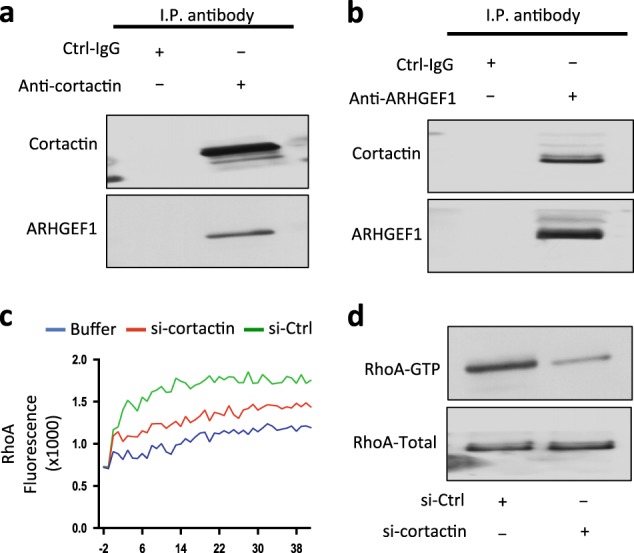
Cortactin associates with ARHGEF1, which undergoes cortactin-dependent activation to enhance activation of RhoA. **a** Immunoblot analysis of anti-cortactin ip or Ctrl-IgG ip, as indicated at the top, using lysates prepared from freshly isolated primary CLL cells; the filters were probed with anti-cortactin or anti-ARHGEF1 antibody, as indicated on the left. **b** Immunoblot analysis of anti-ARHGEF1 ip or Ctrl-IgG ip, as indicated at the top, using lysates prepared from freshly isolated primary CLL cells; filters were probed with anti-cortactin or anti-ARHGEF1 antibody, as indicated on the left. **c** In vitro exchange assay on RhoA of anti-ARHGEF1 ip from lysates of CLL cells transfected with Ctrl-siRNA (green line) or siRNA specific for cortactin (red line) in the presence of Wnt5a. The blue line depicts GTPase-activation using buffer alone. **d** Immunoblot analysis of lysates prepared from MEC1-ROR1 cells transfected 72 h prior with control siRNA or siRNA targeting cortactin; expression of total RhoA, and activated RhoA was measured, as indicated on the left

MEC1-ROR1 cells have higher levels of activated RhoA than MEC1 cells lacking ROR1 [12, 23]. These prior studies showed that ARHGEF1 was recruited to ROR1 and affected activation of RhoA [12, 23]. Here, we performed immunoblot analysis of anti-cortactin or anti-ARHGEF1 ip from lysates of freshly isolated CLL cells, and found that cortactin was associated with ARHGEF1 (Fig. 5a, b). Reducing expression of cortactin via treatment with cortactin siRNA, but not control siRNA, inhibited in the capacity of Wnt5a to induce activation of RhoA by anti-ARHGEF1 ip (Fig. 5c) and reduced the level of activated RhoA in MEC1-ROR1 cells (Fig. 5d), indicating that cortactin contributed to Wnt5a-induced activation of RhoA.

### Proline at position 841 of ROR1 is required for it to associate and activate cortactin

**Fig. 6:**
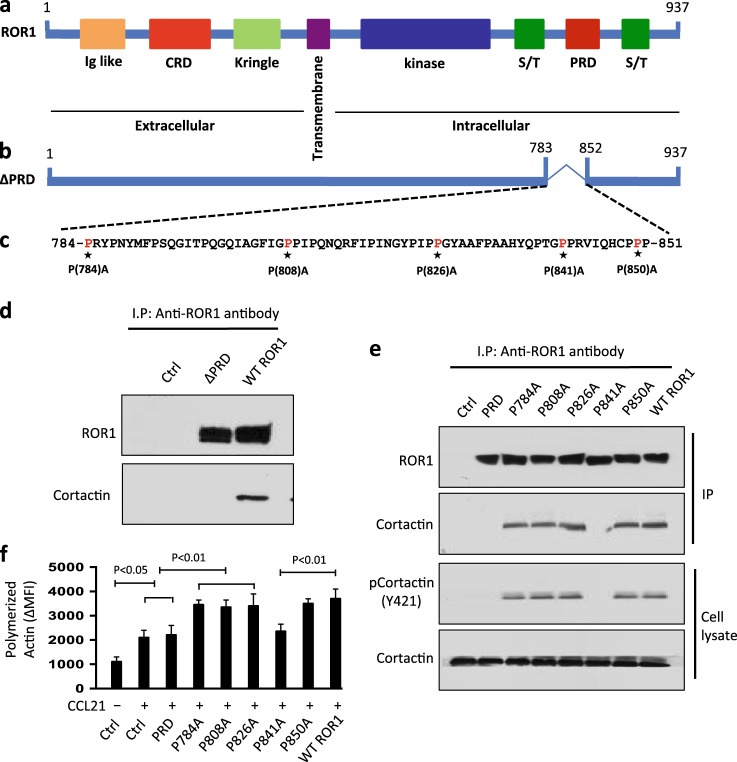
ROR1^P(841)A^ has impaired capacity to associate with cortactin, induce cortactin phosphorylation, or enhance chemokine-directed MEC1-cell F-actin polymerization. **a** Schematic depicts the structure of ROR1 protein with different domains. **b** ΔPRD represents the truncated form of ROR1 without its PRD. **c** Amino-acid sequences of the PRD of ROR1. Asterisks indicate the proline (P) amino-acid residues that had been substituted with alanine (A). **d** Interaction of ROR1 with cortactin was determined by immunoblot analysis of anti-ROR1 ip from lysates of MEC1 (Ctrl), MEC1-ΔPRD, or MEC1-ROR1 (W/T) cells as indicated on the top. **e** Interaction of ROR1 with cortactin was confirmed by immunoblot analysis of anti-ROR1 ip from lysates of MEC1, MEC1-ΔPRD, MEC1-ROR1 (W/T), or MEC1 cells transfected with each of the various mutated forms of ROR1, as indicated on the top (upper panel). In the lower panel is an immunoblot of the whole-cell lysates of the MEC1 (Ctrl), MEC1-ΔPRD, MEC1-ROR1 (W/T), or MEC1 cells transfected with each of the various mutated forms of ROR1, as indicated on the top, and probed with anti-cortactin or anti-pCortactin (Y421) antibody. Expression and tyrosine phosphorylation of cortactin (Y421) was determined in the whole-cell lysates. **f** MEC1 (Ctrl), MEC1-ROR1 (W/T), or MEC1 cells transfected with each of the various mutated forms of ROR1, as indicated at the bottom, were examined for F-actin polymerization in the absence (–) or presence (+) of chemokine CCL21 (100 ng/ml). Data are shown as mean ± S.D. from three independent experiments, (*n* = 3). *P* < 0.05; *P* < 0.01, as calculated using one-way ANOVA with post-hoc Tukey HSD test

In structure cortactin contains a SH3 domain, which can bind to proteins with a PRD such as ROR1, at -P-X-X-P- motifs [1, 16–18]. We examined whether the PRD of ROR1 was required for ROR1 to associate with cortactin. For this, we transfected MEC1 cells with an expression vector encoding wild-type (WT) ROR1 or ∆PRD-ROR1 (ROR1 lacking the PRD) to generate MEC1 cells that both expressed surface ROR1 (Fig. 6a, b). Although the anti-ROR1 ip from lysates of MEC1-W/T ROR1 contained cortactin (Fig. 6d), the anti-ROR1 ip from lysates of MEC1-∆PRD-ROR1 cells did not contain any detectable cortactin (Fig. 6d).

Since the PRD of ROR1 was necessary for ROR1 to associate with cortactin, we generated various mutant forms of ROR1 that had proline (P) to alanine (A) substitutions at positions 784, 808, 826, 841, and 850 within the -P-X-X-P- motifs identified in ROR1-PRD (Fig. 6c). We generated MEC1 cells that each expressed one of these constructs and found they expressed levels of ROR1 and cortactin that were similar to that of MEC1-ROR1, which expressed WT ROR1 (Fig. 6e). ROR1 with *P* = > A substitutions at 784, 808, 826, or 850 could interact and induce phosphorylation of cortactin in MEC1 cells similar to what we noted for W/T ROR1 (Fig. 6e). Moreover, MEC1 cells expressing any one of these mutant forms of ROR1 had enhanced F-actin polymerization in response to CCL21 (Fig. 6f), as did MEC1 cells expressing W/T ROR1. However, ROR1 with a *P* = > A substitution at 841 in the PRD domain of ROR1 (ROR1^P(841)A^) appeared unable to interact with cortactin, or enhance cortactin phosphorylation (Fig. 6e). Moreover, MEC1 cells expressing ROR1^P(841)A^ did not have enhanced chemokine-directed F-actin polymerization, but rather appeared similar to MEC1 cells lacking ROR1 (Fig. 6f).

### Cirmtuzumab, but not ibrutinib, could inhibit Wnt5a-induced activation of cortactin

**Fig. 7:**
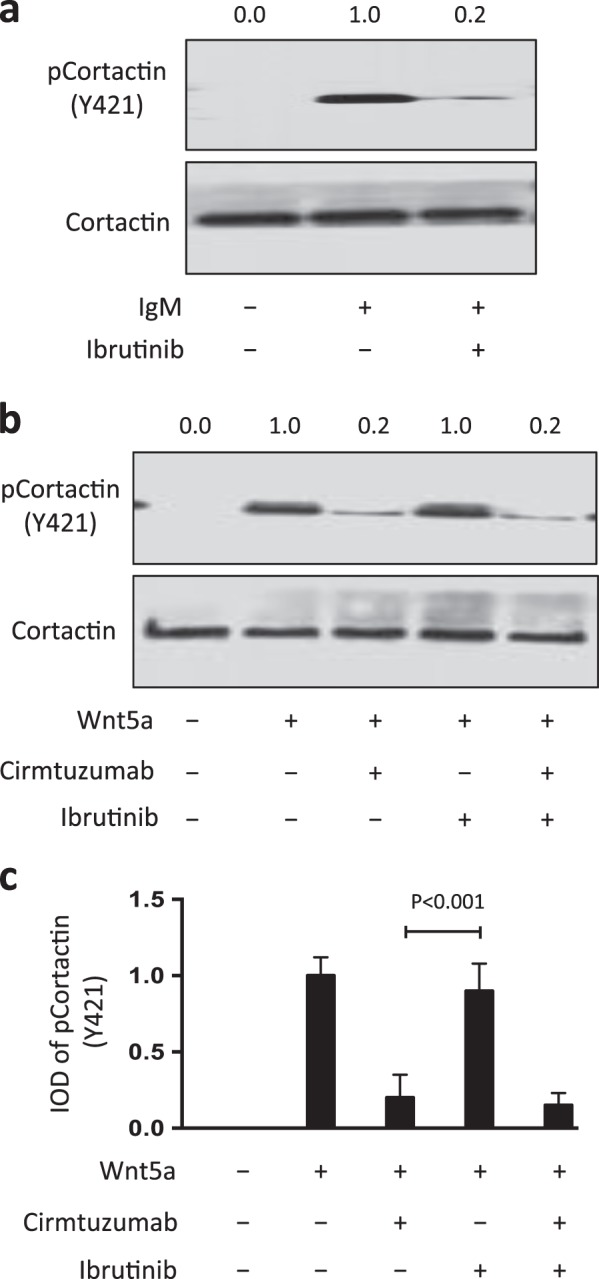
Cirmtuzumab, but not ibrutinib, can inhibit wnt5a-induced activation of cortactin. **a** Immunoblot analysis of lysates prepared from overnight, serum-starved primary CLL cells that subsequently were treated with ibrutinib (0.5 μm), without (–) or with (+) anti-human IgM F(ab)_2_ (10 μg/ml), as indicated at the bottom; the filters were probed with anti-cortactin or anti-pCortactin (Y421) antibody, as indicated on the left. The numbers above each lane are ratios of band IOD of phosphorylated versus total cortactin. **b** Immunoblot analysis of lysates prepared from overnight, serum-starved primary CLL cells that subsequently were treated with cirmtuzumab (10 μg/ml) and/or ibrutinib (0.5 μm), without (–) or with (+) Wnt5a (100 ng/ml), as indicated at the bottom; the filters were probed with anti-cortactin or anti-pCortactin (Y421), as indicated on the left. The numbers above the top lane are ratios of band IOD of phosphorylated cortactin versus total cortactin. **c** Phosphorylation of cortactin (Y421) was measured in serum-starved primary CLL cells that subsequently were treated with cirmtuzumab (10 μg/ml) and/or ibrutinib (0.5 μm), without (–) or with (+) Wnt5a (100 ng/ml), as indicated at the bottom. Data are shown as mean ± SD from three independent experiments, (*n* = 3). *P* < 0.001, as determined by two-tailed Student’s *t-*test

Prior studies found that surface immunoglobulin ligation to induce B-cell-receptor (BCR) signaling could induce phosphorylation of cortactin [21]. We corroborated these findings, observing that BCR signaling induced by anti-IgM (anti-µ) could induce tyrosine phosphorylation of cortactin at Y421 in CLL cells (Fig. 7a). Treatment of CLL cells with ibrutinib, a drug that inhibits BTK and BCR signaling [27], inhibited the capacity of anti-µ to induce cortactin phosphorylation (Fig. 7a); but was unable to inhibit the capacity of Wnt5a to induce phosphorylation of cortactin (Y421) (Fig. 7b, c). On the other hand, cirmtuzumab could block Wnt5a-induced cortactin phosphorylation (Fig. 7b, c).

## Discussion

In the present study we found that Wnt5a induces ROR1 to associate with cortactin, which undergoes tyrosine phosphorylation; this effect could be inhibited by the anti-ROR1 mAb cirmtuzumab. We also found that ROR1/cortactin associated with ARHGEF1, which could activate RhoA. Specific siRNA-mediated silencing of cortactin, or treatment with cirmtuzumab, could inhibit the capacity of Wnt5a to enhance CXCL12-directed CLL-cell migration. Moreover, siRNA-mediated silencing of coractin or treatment with cirmtuzumab, also inhibited the capacity of CLL-cells co-cultured with MSC to have enhanced migration. Collectively, these studies reveal that cortactin plays an important role on ROR1-dependent non-canonical Wnt5a-signaling involved in chemokine-directed migration.

The PRD of ROR1 was necessary for it to interact with cortactin and induce F-actin polymerization in response to Wnt5a. The SH3 domain of cortactin allows it to bind the PRD of other proteins at proline residues within -P-X-X-P- motifs [16–18]. ROR1 with alanine substitutions for proline at position 841 (ROR1^P841A^) did not associate with cortactin in MEC1 cells. Moreover, MEC1 cells transfected with ROR1^P841A^ had levels of phosphorylated cortactin or chemokine-directed F-actin polymerization that were comparable to that of MEC1 cells lacking ROR1, but significantly less than MEC1 cells transfected to express WT ROR1 or mutant ROR1 with proline to alanine substitutions at other -P-X-X-P- motifs within the ROR1-PRD. Thus, the proline residue at 841 of ROR1 appears critical for binding to cortactin, or possibly another cytosolic protein(s), which in turn could interact with cortactin. Therefore, this residue plays an important role for ROR1 to bind and activate cortactin and enhance chemokine-directed F-actin polymerization of MEC1 cells.

Enhanced CLL-cell motility was associated with phosphorylation of cortactin in circulating CLL cells [21]. Other than Wnt5a, cortactin can undergo phosphorylation in response to factors such as the chemokine CXCL12, which can induce chemokine-receptor signaling via CXCR4, or by anti-µ, which could induce BCR signaling [21]. However, the results of our study suggest that Wnt5a also may induce cortactin phosphorylation in CLL cells via ROR1-dependent signaling [12]. Culturing CLL cells in media lacking Wnt5a resulted in rapid reduction in the levels of phosphorylated cortactin, which could be restored by treatment of CLL cells with exogenous Wnt5a. Similarly, we found that MEC1 cells have undetectable levels of phosphorylated cortactin unless they are transfected to express ROR1, which allows for ROR1 signaling in response to autocrine Wnt5a. Ibrutinib, at drug concentrations that could block BTK and BCR signaling [27, 28], could not inhibit the capacity of Wnt5a to induce cortactin phosphorylation (Y421) or enhance CLL-cell motility.

In a prior study, we found that Wnt5a could induce activation of RhoA via the recruitment and activation of ARHGEF1 to ROR1 [12]. We also found that HS1 could function as an adaptor protein for the recruitment of ARHGEF1 to ROR1 and could contribute to Wnt5a-enhanced migration of CLL cells [23]. In the present study we found that cortactin also can interact with ROR1 and activate ARHGEF1 to enhance activation of RhoA and chemokine-directed cell migration [29]. Consistent with the notion that cortactin plays an important role in such signaling is our finding that leukemia cells with reduced expression of cortactin following treatment with specific siRNA were significantly less responsive to Wnt5a, indicating that cortactin contributes to ROR1-signaling leading to enhanced leukemia-cell migration. Moreover, silencing of both cortactin and HS1 in CLL cells had an additive effect on the inhibition of Wnt5a-enhanced chemokine-directed migration that was significantly greater than that achieved by silencing either cortactin or HS1 alone. This indicates that both cortactin and HS1 are involved in the capacity of Wnt5a to enhance leukemia-cell motility and that the deficiency of one cannot be fully compensated by the other. Collectively, these studies reveal that Wnt5a induces ROR1 to complex with cortactin/HS1/ARHGEF1 at P841, which induces phosphorylation of cortactin and HS1, activation of ARHGEF1 and RhoA, thereby enhancing leukemia-cell migration.

Surely, cortactin may facilitate motility of cells that do not express ROR1, possibly by its capacity to associate with other cell surface receptors, which induce actin-cytoskeleton reorganization necessary for cell migration [13, 18, 30–33]. Indeed, cortactin can facilitate F-actin polymerization with the association of the Arp2/3 complex [34, 35]. Mice deficient in cortactin have impaired leukocyte transmigration and defective neutrophil extravasation [36]. Moreover, cells deficient in cortactin had impaired motility due to defects in the persistence of lamellipodial protrusions [37]. In those cases, cortactin contributes to such cellular functions by interacting with other binding partners following stimulation of surface receptors other than ROR1, such as CXCR4 or CCR7 [13, 14, 18, 30–33, 38]. Nevertheless, our studies show that cortactin can interact with ROR1 and enhance migration of CLL cells that express ROR1.

In summary, the present study describes a previously unrecognized activation of RhoA via ROR1/cortactin/ARHGEF1-dependent mechanism in response to Wnt5a, which prior studies found is present at high levels in the plasma of patients with CLL and is presumably present at even higher levels in lymphoid tissues. Conceivably this could enhance leukemia-cell migration to and retention within the lymph nodes, allowing CLL cells' greater opportunity to receive additional growth/survival factors from accessory cells within the leukemia microenvironment. The reported findings highlight a pathway for potential drug development involving the importance of cortactin in ROR1-dependent Wnt5a-induced signaling. To this end, we found that the capacity of Wnt5a to induce ROR1 to interact and activate cortactin, recruit and activate ARHGEF1, and enhance leukemia-cell migration could be blocked by cirmtuzumab, a first-in-class humanized anti-ROR1 mAb, which is being evaluated in patients with CLL (https://clinicaltrials.gov/ct2/show/NCT02222688) [39, 40]. Taken together, these studies demonstrate that cirmtuzumab can inhibit activation of ROR1-dependent growth/survival/migration-signaling pathways that appear unaffected by treatment with ibrutinib [28], supporting the rationale for clinical evaluation of this antibody alone or in combination with other targeted therapies in patients with CLL.

## Electronic supplementary material

Supplementary Information

Supplementary Figures
