# Supplementary material for: Wnt5a causes ROR1 to complex and activate cortactin to enhance migration of chronic lymphocytic leukemia cells
Source: Leukemia. 2018 Dec 19;33(3):653–61. doi: 10.1038/s41375-018-0306-7 (PMC6462876; doi:10.1038/s41375-018-0306-7)
Supplement: Supplementary file 1 — Supplementary Information [file 41375_2018_306_MOESM1_ESM.docx]

**supplementary information**

**Wnt5a Causes ROR1 To Complex and Activate Cortactin To Enhance Migration Of Chronic Lymphocytic Leukemia Cells**

**Keywords:** Wnt5a, ROR1, Cortactin, Cell Migration, Chronic Lymphocytic Leukemia

**Running Title:** Wnt5a causes ROR1 to complex with cortactin

**Supplementary Materials and Methods**

**Immunoprecipitation analysis**

Immunoprecipitation analysis was performed as described.^1^ Cells were lysed in a buffer containing 1% Nonidet P-40, 10 mM Tris-HCl (pH 7.5), 50 mM NaCl, and 1 mM EDTA with protease inhibitors (Roche). The lysates were cleared by centrifugation at 16,000 x *g* for 15 minutes. Immune precipitates (ip) were isolated using protein A agarose beads, followed by immunoblot or MS analysis. Antibodies for immune precipitation were as follows: the anti-ROR1 antibodies (cirmtuzumab or 4A5) were generated in our laboratory; the anti-cortactin or ARHGEF1 antibody was obtained from Cell Signaling Technology.

**Immunoblot analysis**

Western blot analysis was performed as described.^1^ Equal amounts of total protein from each sample were separated by SDS-PAGE and blotted onto polyvinylidene difluoride membrane (filter). Western blot analysis was performed using primary mAbs specific for ROR1, cortactin, pCortactin (Y421), ARHGEF1 or β-actin, which were detected using secondary antibodies conjugated with horseradish peroxidase. All antibodies were purchased from Cell Signaling Technology, Danvers, MA, USA.

**Cell migration assay**

The cell migration assay was preformed as described.^2, 3^ Briefly, A total of 5 × 10^5^ cells were washed twice, cultured overnight in serum-free medium, and then treated with or without Wnt5a (200 ng/ml) for 30 minutes. The cells then were placed into the top chamber of a Transwell culture polycarbonate insert with 6.5-mm diameter and 5 μm of pore size (Corning). Cells were incubated for 2 hours in serum-free medium at 37°C and 5% CO_2_, and the migration toward chemokine (CXCL12, 200 ng/ml or CCL21, 200 ng/ml) was analyzed by flow cytometry. The percentage of migrating cells was calculated as the number of migrated cells in response to chemokine divided by the total number of input cells.

**Site-specific mutation**

Site-specific mutations were performed as described previously.^4^ In brief, mutation constructs were generated with QuikChange Site-Directed Mutagenesis System (Invitrogen) on the basis of the parental construct (wild-type ROR1), according to the manufacturer's instructions. The mutations were verified by DNA sequencing.

The following primer sets were used:

P(784)A, 5'-CAGTGAGTAATCTCAGTAACGCCAGATATC-3' (sense) and 5'-CATGTAATTAGGATATCTGGCGTTACTGAG-3' (antisense);

P(808)A, 5'-GATTGCTGGTTTCATTGGCGCGCCAATACC-3' (sense) and 5'-GGTTCTGAGGTATTGGCGCGCCAATGAAACC-3' (antisense);

P(826)A 5'-CAATGGATACCCAATACCTGCTGGATATGCAGC-3' (sense) and 5'-GGAAACGCTGCATATCCAGCAGGTATTGG-3' (antisense);

P(841)A, 5-CCAGCCAACAGGTGCTCCCAGAGTGATTC-3 (sense) and 5-GCTGAATCACTCTGGGAGCACCTGTTGG-3 (antisense);

P(850)A, 5-GTGATTCAGCACTGCCCAGCTCCCAAGAG-3 (sense) and 5-CGACTCTTGGGAGCTGGGCAGTGCTG-3 (antisense).

**Nucleofection of plasmids and siRNAs**

Human B Cell Nucleofector Kit for siRNA or plasmid transfection was from Lonza. B-CLL cells or MEC1 cells (5 × 106) were suspended in 100 μl Nucleofector Solution with plasmids or siRNAs (Life Technologies) and transfected with the Nucleofector II device (program U-015). The transfected cells were cultured in 12-well plates in complete medium for 48 hours (plasmids) or 72 hours (siRNAs) and then subjected to immunoblot analysis and assays.

**Co-culture migration assay**

To collect mesenchymal stromal cells (MSCs) conditioned medium (MSC-CM), MSCs isolated from bone marrow of CLL patients were cultured at 37°C in a humidified atmosphere containing 5% O_2_ for 3 days that constitutively secrete CXCL12, or Wnt5a as described previously.^5, 6^ A total of 5 × 10^5^ CLL cells were washed twice, and then were placed into the top chamber of a Transwell culture polycarbonate insert with 6.5-mm diameter and 5 μm of pore size (Corning). Cells were incubated for 2 hours, and the migration toward MSC-CM was analyzed by flow cytometry. The percentage of migrating cells was calculated as the number of migrated cells in response to MSC-CM divided by the total number of input cells.

**Actin polymerization assay**

F-actin polymerization assay was performed as described.^3^ Briefly, cells (1 × 10^6^/mL) were suspended in RPMI-1640 medium with 0.5% BSA and then stimulated in the presence or absence of CCL21 (100 ng/ml) for 15 sec at 37 °C. 400 μL of the cell suspension were added to 100 μL of assay buffer containing 4 × 10^−7^ mol/L FITC-labeled phalloidin (Thermo Fisher Scientific), 0.5 mg/mL 1-α-lysophosphatidylcholine (Sigma), and 4% formaldehyde in phosphate-buffered saline (PBS) for 15 min. Fixed cells were analyzed by flowcytometry on a FACSCanto II (BD Biosciences), and the delta mean fluorescence intensity (ΔMFI) was determined for each sample, which was calculated by subtracting the intensity of control stained cells from that of stained cells.

**RhoGEF nucleotide exchange activity assay**

RhoGEF exchange assay kit was from Cytoskeleton and used as per the manufacturer’s instructions. For *in-vitro* guanine nucleotide exchange activity on RhoA, ARHGEF1 was immunoprecipitated from CLL cells that previously transfected with si-Ctrl or si-cortactin. Reactions were measured in a Tecan Spectrofluor plus fluorimeter (λ_ex_=360nm, λ_em_=460nm). Pull-downed ARHGEF1 were added after 120 seconds. Readings were taken at 20°C every 1 min for a total reaction time of 44 minutes. The exchange curve can be achieved by exporting raw data to Microsoft Excel and analyzing the data using GraphPad Prism 6.0.

**RhoA activation assay**

RhoA activation assay reagents were purchased from Cytoskeleton and used per the manufacturer’s instructions. Briefly, GTP-bound active RhoA was pulled down with Rhotekin-RBD beads for 1 hour at 4 °C, and then subjected to immunoblot analysis. Immunoblots of whole-cell lysates were used to assess for total RhoA.

**Study approval**

Blood samples were collected from CLL patients at the Moores Cancer Center who satisfied diagnostic and immuno-phenotypic criteria for common B cell CLL and who provided written, informed consent, in compliance with the Declaration of Helsinki and the Institutional Review Board (IRB) of the UCSD (IRB approval number 080918).

**Supplementary Figure Legends**

**Supplementary Figure S1.** Immunoblot analysis of lysates prepared from freshly-isolated primary CLL cells that were serum-starved for the times indicated on the top (in hours); the filters were probed with anti-cortactin, or anti-phospho cortactin (Y421), as indicated on the left.

**Supplementary Figure S2.** (**A)** CLL cells transfected 72-hours previously with control siRNA or siRNA targeting cortactin, and cell migration in response to MSC-CM was assessed, as indicated at the bottom. Data are shown as mean ± SD from 3 independent experiments of CLL cells from each of 6 patients. *P*<0.01; *P*<0.001, as assessed by 2-tailed Student’s *t* test. (**B**) Migration capacity of CLL cells treated with Ctrl-IgG or cirmtuzumab (10 μg/ml), was measured in response to MSC-CM as indicated at the bottom. Data are shown as mean ± SD from 3 independent experiments of CLL cells from each of 6 patients. *P*<0.01; *P*<0.001, as assessed by 2-tailed Student’s *t* test.

**Supplementary Figure S3.** CLL cells were transfected 72-hours previously with control siRNA or siRNA targeting cortactin, or HS1, or both cortactin and HS1; and cell migration in response to CXCL12 (200 ng/ml) was examined without (–) or with (+) addition of exogenous Wnt5a (200 ng/ml), as indicated at the bottom. Data are shown as mean ± SD from 3 independent experiments of CLL cells from each of 6 patients. *P* < 0.05; *P* < 0.01; *P* < 0.001, as assessed by 1-way ANOVA with post-hoc Tukey HSD test.

**Supplementary References**

1. Widhopf GF, 2nd, Cui B, Ghia EM, Chen L, Messer K, Shen Z*, et al.* ROR1 can interact with TCL1 and enhance leukemogenesis in Emu-TCL1 transgenic mice. *Proc Natl Acad Sci U S A* 2014 Jan 14; **111**(2)**:** 793-798.

2. Yu J, Chen L, Cui B, Widhopf GF, 2nd, Shen Z, Wu R*, et al.* Wnt5a induces ROR1/ROR2 heterooligomerization to enhance leukemia chemotaxis and proliferation. *J Clin Invest* 2015 Dec 21.

3. Burger JA, Burger M, Kipps TJ. Chronic lymphocytic leukemia B cells express functional CXCR4 chemokine receptors that mediate spontaneous migration beneath bone marrow stromal cells. *Blood* 1999 Dec 01; **94**(11)**:** 3658-3667.

4. Hasan MK, Nafady A, Takatori A, Kishida S, Ohira M, Suenaga Y*, et al.* ALK is a MYCN target gene and regulates cell migration and invasion in neuroblastoma. *Sci Rep* 2013 Dec 20; **3:** 3450.

5. Fecteau JF, Messmer D, Zhang S, Cui B, Chen L, Kipps TJ. Impact of oxygen concentration on growth of mesenchymal stromal cells from the marrow of patients with chronic lymphocytic leukemia. *Blood* 2013 Feb 7; **121**(6)**:** 971-974.

6. Fecteau JF, Kipps TJ. Structure and function of the hematopoietic cancer niche: focus on chronic lymphocytic leukemia. *Front Biosci (Schol Ed)* 2012 Jan 1; **4:** 61-73.
