# Supplementary figures and images for: Wnt5a causes ROR1 to complex and activate cortactin to enhance migration of chronic lymphocytic leukemia cells
Source: Leukemia. 2018 Dec 19;33(3):653–61. doi: 10.1038/s41375-018-0306-7 (PMC6462876; doi:10.1038/s41375-018-0306-7)

Supplementary Figure S1

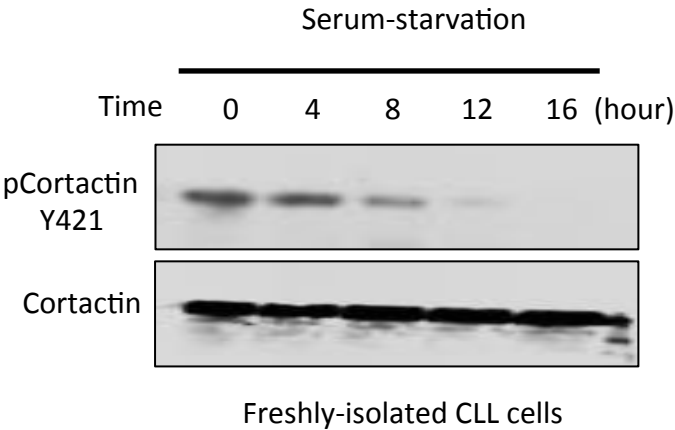

## Supplementary Figure S2

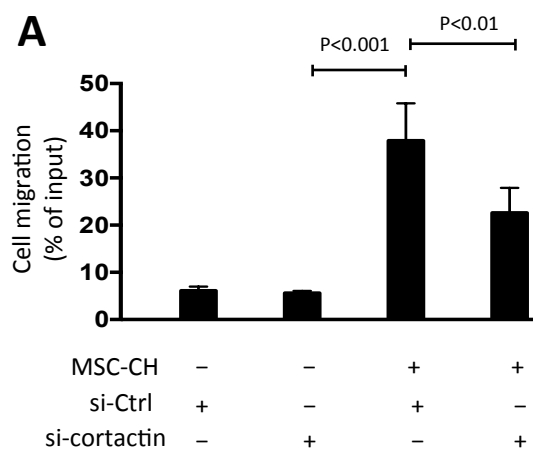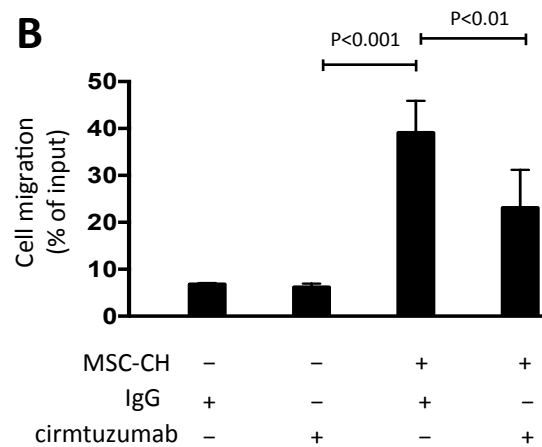

Supplementary Figure S3

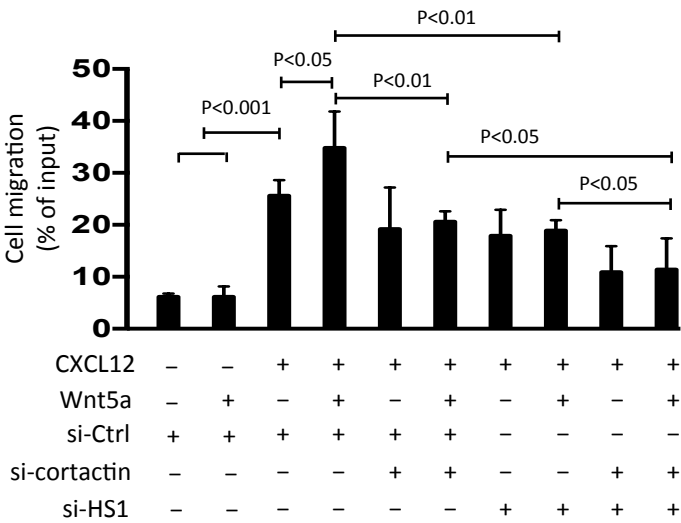

Supplement: Supplementary file 2 — Supplementary Figures [file 41375_2018_306_MOESM2_ESM.pdf]
